# ASSESSING NUTRITIONAL RISK IN ADULT DAY SERVICES: UTILITY OF THE DETERMINE CHECKLIST

**DOI:** 10.1093/geroni/igz038.838

**Published:** 2019-11-08

**Authors:** Shannon E Jarrott

**Affiliations:** The Ohio State University, Columbus, Ohio, United States

## Abstract

Older adults attending adult day services (ADS) often possess risk factors for malnutrition, such as chronic disease, physical disability, and cognitive impairment. We explored the utility of administering to ADS participants the DETERMINE Checklist - a measure of nutritional status. Among eleven participants (M age=77.3 years), 82% (n = 9) presented high nutritional risk. The three most common risk factors were: difficulty shopping, cooking, and/or feeding themselves (100%), making health-related dietary changes (63.7%), and taking three or more daily medications (63.7%). Our preliminary findings indicate that ADS participants may be at moderate-high risk of malnutrition; however, the DETERMINE Checklist may require modification for an ADS population. For example, the checklist may be more reliable if completed jointly by a participant and informal caregiver. We present recommendations for adaptions based on our pilot data as well as implications for ADS staff and clinicians.

